# Permeation of a Range of Species through Polymer Layers under Varying Conditions of Temperature and Pressure: In Situ Measurement Methods

**DOI:** 10.3390/polym11061056

**Published:** 2019-06-17

**Authors:** Bernadette Craster, Timothy G.J. Jones

**Affiliations:** 1 TWI Ltd, Granta Park, Great Abington, Cambridge CB21 6AL, UK; 2 Schlumberger Cambridge Research, High Cross, Madingley Rd, Cambridge CB3 0EL, UK; jones49@slb.com

**Keywords:** Supercritical carbon dioxide, hydrogen sulfide, permeation testing at temperature and pressure, in situ measurement, electrolyte transport, moulded polymers, polyether ether ketone, polyphenylene sulfide

## Abstract

Minimising the transport of corrosive reactants such as carbon dioxide, hydrogen sulfide and chloride ions to the surfaces of carbon steel pipes by the use of polymer barrier layers is of major interest in the oil and gas sector. In these applications, there is a requirement to assess the performance of these barrier layers although it is difficult to perform long-term predictions of barrier properties from the results of short-term measurements. New methodologies have been successfully developed to study the permeability of carbon dioxide (CO_2_) and hydrogen sulfide (H_2_S) through polymer layers under variable conditions of elevated temperatures of 100 °C and pressures of the order of 400 barg. In situ variation of the temperature and the inlet pressure of the gas (or gas mixture) allowed the activation energy and pressure dependence of the permeability to be determined without outgassing of the specimen. These methodologies have been applied to the measurement of the permeability of moulded polyphenylene sulfide (PPS) to supercritical CO_2_ in the presence of H_2_S. The diffusion coefficients of sodium chloride and potassium chloride through both PPS and polyether ether ketone (PEEK) at ambient temperature and pressure have also been measured.

## 1. Introduction

The continuing demand for oil and gas means they are being sourced from extremely corrosive environments, resulting in increased production costs [1,2]. In this operating environment the chemical species that accelerate corrosion and stress corrosion include carbon dioxide (CO_2_), hydrogen sulfide (H_2_S) and chloride ions (Cl^−^). As an example, the carbon dioxide and hydrogen sulfide may be combined in a supercritical mixture at elevated temperature and pressure. The chloride ions will be present in sea water at near ambient temperatures on the outside of flow lines and likely to be present in produced water at elevated temperature inside the flow lines [3]. The pressure in a flexible riser or flow line varies depending on the location but can be as high as 600 barg with temperatures varying up to 200 °C. 

Industry methods to mitigate corrosion in the presence of these fluids include the use of corrosion resistant alloys, corrosion inhibitors and moulded polymer layers in installations such as flexible risers, flow lines and polymer lined pipes [4]. Minimising the transport of corrosive reactants to a carbon steel pipe surface by the use of polymer barrier layers is of major interest in the oil and gas sector [5,6]. However, in these applications, there is a requirement to assess the performance of these barrier layers, although it is difficult to perform long-term predictions of barrier properties from the results of short-term measurements [7].

There already exists a research interest in short-term, single-condition permeation testing at elevated pressures for the oil and gas industry, encapsulation and gas separation processes and for lower pressure applications such as packaging for food and pharmaceuticals.

In the oil and gas industry, methods to determine the transport levels of single gases or mixtures of methane, carbon dioxide and hydrogen sulfide through polymers such as polyethylene, various polyamides, polyvinylidene fluorides, and elastomers have been proposed and validated. Generally these methods are based on manometric measurements where the flux of single components are inferred from pressure measurements. Transport and related coefficients such as permeability, solubility and diffusion have previously been determined, [8,9,10,11,12,13]. Heilman et al. [14] measured the permeability and diffusivity coefficients of fluorinated polymers, polyamides, polyethylene terephthalate, and polyvinylchloride using closed-cell manometric methods with a maximum inlet pressure of 1.011 bar and a maximum temperature of 80 °C. Heilman et al. [14] measured values of the permeability coefficient in the range of 6 × 10^−12^ cm^2^ s^−1^ bar^−1^ for cellophane and 4.3 × 10^−8^ cm^2^ s^−1^ bar^−1^ for polyethylene in the temperature range 30–45 °C. Stern and Bhide [15] used a flow method at pressures of 7.5 bar and a maximum temperature of 55 °C to measure the permeability coefficient of hydrogen sulfide through a range of silicone polymers, obtaining values in the range 8 × 10^−7^ to 8 × 10^−5^ cm^2^ s^−1^ bar^−1^. Merkel and Toy [16] measured the permeability coefficients of hydrogen sulfide and carbon dioxide through both fluorinated and nonfluorinated polymers; measured values of the permeability coefficient to carbon dioxide ranged from 8 × 10^−10^ cm^2^ s^−1^ bar^−1^ for Nylon to 8 × 10^−5^ cm^2^ s^−1^ bar^−1^ for polytetrafluoroethylene (PTFE). Flaconnèche et al. [17] used a continuous flow permeation method to determine the transport of CH_4_–CO_2_ gas mixtures through polyethylene and polyvinylidene at pressures up to 100 bar.

Nilsson et al. [18] measured the permeability, solubility and diffusion coefficients of nitrogen gas in polycarbonate and polyether ether ketone polymers at a pressure of 670 bar and at temperatures in the range of 100 to 250 °C. In contrast, Celina and Gillen [19] used a continuous flow method for measuring the permeability of oxygen through elastomers at pressures of approximately 315 mm Hg and temperatures of 225 °C.

The separation processes in gas membranes have been studied using permeation test equipment at elevated temperatures and a range of pressures [20,21,22,23,24,25,26]. These methods also rely on pressure measurement to monitor the gas transport. In the food packaging industry, the transport of oxygen and water can reduce the shelf life of the food product and there is a need to have test methods available to study the barrier properties of thin films [27,28,29,30,31]. Manometric permeation tests were carried out at various intervals up to a period of 200 days by Huang and Paul [32] and Rowe et al. [33]. Huang and Paul [32] measured the decrease in the permeability coefficients of very thin films of the glassy polymers polysulfone, polyimide and poly(2,6-dimethyl-1,4-phenylene oxide) at a temperature of 35 °C and a pressure differential of 2 bar to the gases methane, oxygen and nitrogen to determine the changes in their selectivity as gas separation membranes over these long time periods. In another study by Bernardo et al. [34], the permeability of polymers of intrinsic microporosity (PIMS) to combinations of CO_2_, CH_4_, N_2_ and O_2_ was determined. The specimens were removed from an ageing environment and placed into the permeation cell at periodic intervals over a period of four years. 

The transport of chloride ions through the moulded polymeric protective layers has already been mentioned as a potential source of corrosion. Extensive studies have been carried out on the transport of organic and inorganic salts through swollen membranes in order to determine rejection coefficients and these provide useful information about data treatment [35,36,37]. Extensive studies have been carried out on the transport of water and salts through swollen polymer membranes used in desalination, ion exchange and the polymer membranes used in fuel cells [35,36,37,38,39]. In contrast, there are few studies on the diffusion of salt through moulded polymer layers within the oil and gas literature. Limited information can be drawn from experiments on the transport of salt in the polymers of different composition used under different conditions. Roe et al. [40] and Valadão et al. [41] measured the diffusion of sodium chloride in moulded high-density polyethylene (HDPE) geomembranes under conditions of ambient temperature and pressure, yielding values of less than 6 × 10^−11^ cm^2^ s^−1^ [33] and 1.5 × 10^−8^ cm^2^ s^−1^ [41]. The transport of water and salts in elastomers has attracted greater interest. Harogoppad et al. [37] and Harogoppad and Aminabhavi [42] measured the permeability and diffusivity of a range of salts in polyurethane membranes over the temperature range 25–60 °C, giving values of the permeability coefficient of inorganic salts at low concentration in the range 3.7–5.5 × 10^−9^ cm^2^ s^−1^ at 25 °C. Cassidy and Aminabhavi [43], Aminabhavi et al. [44] and Aithal et al. [45] measured the rates of permeation of water and sodium chloride solutions for a range of elastomers, including nitrile butadiene rubber (NBR) and ethylene propylene diene monomer (EPDM) rubber over the temperature range 23–60 °C. Aithal et al. [45] reported values of the diffusion coefficient for sodium chloride in four different rubbers to be in the range 4.1–5.6 × 10^−14^ cm^2^ s^−1^ at a temperature of 23 °C. Experimental methods for determining the permeability and diffusion coefficients of salts through membranes and modified clay films have been described [38,46,47,48].

The principal objective of this paper is to describe the measurement of the permeabilities and diffusion coefficients of gases in polymer membranes at elevated temperatures and pressures over timescales of several months. During these long-term measurements, changes in the test conditions, principally temperature and differential gas pressure, were made to simulate the changes in operating conditions that the polymers experience in use. The polymers chosen for this study were polyphenylene sulfide (PPS) and polyether ether ketone (PEEK), which are representative of the high-performance thermoplastics that are increasingly being proposed as barrier layers for metallic components deployed in the harsh conditions of producing oil and gas wells. 

The testing of polymer membranes to determine permeability and diffusion coefficients and gas solubilities, with in situ changes in temperature and inlet gas pressure, over time scales of many months requires the development of new methodologies. Measuring the changes in the permeability of polymer membranes as a function of temperature and inlet pressure can yield information on polymer degradation (e.g., in the presence of hydrogen sulfide) and changes in mechanical properties, which can provide input data to models for predictions of barrier lifetime [49]. 

### 1.1. Introduction to Transport through Polymers

The internal structure of polymers has a significant influence on the permeation rate of solutes [35,37,50,51,52]. An amorphous thermoplastic, for example, has an irregular and random distribution of polymer chains resulting in “free space” or free volume within the structure. This free space would allow gases and hydrated ions to permeate relatively quickly. For example, McKeen [53] reported a permeability coefficient twice as large for CO_2_ through amorphous PEEK thin films compared with a crystalline sample. Similarly, Cowling and Park [54] studied the transport rate of H_2_, Ne, N_2_ and CO_2_ through a mixed cis and trans 1,4-polybutadiene membrane, and one consisting of 90% trans 1,4-polybutadiene, the latter being described as crystalline. The permeability of all species was approximately one order of magnitude greater for the largely amorphous mixed cis and trans 1,4-polybutadiene. The degree of crystallinity and the extent of the free volume in the amorphous region can be altered by straining specimens. Yasuda et al. [55] developed a special permeation cell that allowed natural rubber, polyethylene and polypropylene films to be strained during the test. Subsequently, it was found that the transport rates of CO_2_, O_2_, H_2_O, C_2_H_6_ and He through polyethylene and polypropylene increased on straining by a maximum of eight percent, while the permeability through the rubber sample remained unchanged. In contrast, Yasuda and Peterlin [56] studied the permeability of previously drawn low-density polyethylene films to CO_2_ and confirmed that after straining by several hundred percent, the permeability decreased. Studies by Sha and Harrison [57] showed that the permeability of CO_2_ in HDPE was reduced by two orders of magnitude after previously being strained to a draw ratio of 15. 

The amorphous region of a polymer can be rearranged as the temperature is increased above the glass transition temperature (*T*_g_). The fractional volume of voids in the amorphous region increases linearly with temperature above *T*_g_. The importance of *T*_g_ for some engineering polymers is that it determines the maximum temperature of use, particularly for load-bearing structures and those required to exhibit collapse resistance. There is some indication that *T*_g_ can be altered during exposure to fluids, which may be considered as part of the polymer’s ageing process. A common occurrence is that the exposure of polymers to supercritical CO_2_ can alter their *T*_g_, crystallisation temperature and degree of crystallisation at their operational temperature. Kikic et al. [58] studied the reduction in *T*_g_ of poly(2,6 dimethyl phenylene oxide) PPO, poly(acrylic acid) (PAA) and the copolymer vinylpyrrolidene-vinyl acetate, P(VP–VA) during the plasticisation by supercritical CO_2_. Kikic et al. [58] explained the decrease in *T*_g_ on exposure to supercritical CO_2_ by a combination of the swelling of the polymers and the dissolved CO_2_ acting as a lubricant to facilitate the relative motion (sliding) of the polymer chains. Bologna et al. [59] reported the increased crystallinity in amorphous polycarbonate (PC) and PEEK films on heating in the presence of supercritical CO_2_. 

There have been a number of reports that the dependence of the diffusion and permeability coefficients on temperature changes at *T*_g_ [54,60,61]. In a study of transport of O_2_ and N_2_ through poly (4 methyl pentene-1). Kumazawa et al. [62] observed changes in the slope of the Arrhenius style plots of permeability versus pressure at a temperature of 34 °C, which was close to the reported *T*_g_ of 40 °C; the activation energy for gas permeation was larger at temperatures above *T*_g_. In a study of argon permeation through poly(vinyl acetate), which had a measured *T*_g_ of 32 °C, slight discontinuities or changes in slope were detected at 28.65 and 16.69 °C in the Arrhenius plot for the diffusion coefficient [63]. The activation energy for gas diffusion was successively higher at temperatures below the temperatures at which the discontinuities occurred. Yampolskii et al. [64] carried out experiments to determine the solubility coefficients of argon and CO_2_ in poly(trimethylsilyl norbornene) as a function of temperature and discontinuities were observed at 109 and 106 °C, respectively, which were close to the measured *T*_g_ of 104 °C. It is of interest to determine if changes in the value of *T*_g_ (e.g., as a result of aging or plasticisation of the polymer), can be determined during long-term permeation testing by performing temperature sweeps. 

The other parameter that can affect the transport rate is the applied pressure. The literature indicates that the permeability of a polymer to gas may remain constant with feed pressure, increase or, in some cases, decrease [50,65,66,67,68]. These complex and different trends justify the need for a pressure variation study for each polymer and fluid combination.

As polymers age their transport coefficients can be altered [33,34]. Ageing of a polymer can result in alterations in the internal arrangements of crystalline and amorphous phases as well as the removal of plasticisers. These internal changes are often obvious from the slope of the plateau on the permeation trace if the test is allowed to continue for a reasonable duration. A literature search did not reveal any published study on the use of long-term permeation tests to monitor ageing in polymers at elevated temperatures and pressures. 

### 1.2. Gas Transport through Polymers

Any study of the transport of mixed gases through a polymer to the steel surface requires reliable experimental methods that allow the individual transported components to be quantified. The continuous flow method is used as a methodology to measure the transport of components of gas mixtures through membranes [20,69]. In this experiment, a gas mixture is pressurised on the face of a polymer film that is supported underneath by a porous steel frit. On the underside of the polymer film, a nitrogen sweep of a known flow rate carries the permeated gases to a gas chromatograph to be quantified. The permeability *K_i_* for species *i* is calculated using

(1) $$K_{i}=\frac{Q_{max,i}l}{A\Delta P_{i}}$$

where *Q_max_,_i_* is the maximum volume flow rate (cm^3^ (STP) s^−1^), *l* the sample thickness (cm), *A* the surface area (cm^2^) through which permeation occurs and **Δ*P_i_*** (barg) is the difference in the partial pressure of the species *i* in the initial gas mixture. In some circumstances, *P_i_* can be replaced by a fugacity term reflecting the nonideality of the test fluid [66,70,71]. The fugacity can be calculated using NIST REFPROP standard reference database 23 version 9. 

The permeability *K_i_* (cm^3^ (STP) cm^−1^ s^−1^ bar^−1^) of a dense polymer to a species *i* depends on transit time or diffusion coefficient, *D_i_* (cm^2^ s^−1^) and the solubility coefficient *S_i_* (barg^−1^), or affinity for the internal surface. The relationship is described by

(2) $$K_{i}=D_{i}S_{i}$$

In most cases, Henry’s law is applicable, namely,

(3) $$C_{eqm}=P_{i}S_{i}$$

where the equilibrium concentration, *C_eqm_*, (g of gas per g of polymer) of a gas dissolved in a polymer varies linearly with its partial pressure *P_i_* [69]. Again, fugacity can be used in place of partial pressure [71].

For materials where the permeation process does not vary with the concentration of dissolved species, then the diffusion coefficient for species *i*, *D_i_*, can be calculated by the time lag method [69,72,73,74], using

(4) $$Q_{acc,i}=\frac{D_{i}C_{i}}{l}\left(t-\frac{l^{2}}{6D_{i}}\right)$$

where, *Q_acc.i_* is the cumulative mass of species *i* per unit area that has diffused through the polymer. This value of *Q_acc.i_* can be calculated by integrating the flux (*Q*) measured at the gas chromatograph as a function of time and *D_i_* can be calculated from:

(5) $$D_{i}=\frac{l^{2}}{6\theta }$$

where, θ, the time lag (*s*), is the intercept in a plot of accumulated volume of permeant *Q_acc.i_* versus time. 

For the materials under test, the value of *K_i_* is expected to change exponentially as the temperature increases:

(6) $$K_{i}=K_{0}\exp\left(-\frac{E_{ak}}{RT}\right)$$

where *K_0_* is a constant, *E_ak_* (kJ mol^−1^) is the activation energy for permeation to occur, *R* (kJ mol^−1^ K^−1^) is the ideal gas constant and *T* (K) is absolute temperature [19,50].

A similar relationship holds for the diffusion coefficient

(7) $$D_{i}=D_{0}\exp\left(-\frac{E_{aD}}{RT}\right)$$

where *E_ak_* and *E_aD_* are related, as a consequence of Equation (2), by

(8) $$E_{aK}=E_{aD}+\Delta H$$

and Δ*H* (kJ mol^−1^) is the enthalpy of sorption [51]. It should be noted that *E_ak_* can be positive or negative since although *E_aD_* is always positive, Δ*H* can be either positive or negative. The diffusion coefficient *D* can be related to the fractional free volume (*FFV*) by an equation of the form [51],

(9) $$D=A_{0}\exp\left(-\frac{B}{FFV}\right)$$

where *A_o_* and *B* are empirical constants. Combining Equations (7) and (9) relates the *E_aD_* to *FFV* by an equation of the form

(10) $$E_{aD}=RT\left(A^{\prime }+\frac{B}{FFV}\right)$$

where *A’* is *lnD_0_-lnA_o_*. Monitoring the activation energy for diffusion *E_aD_* over time enables changes in *FFV* to be determined and possibly allow changes in the mechanical properties of polymeric materials to be inferred. 

As mentioned above, a review of the literature indicates that the permeability of a polymer to gas may remain constant with feed pressure, increase or in some cases decrease [52,59,66,69]. Essentially the response depends on the alteration in the diffusion coefficient with the supply concentration and/or the alteration in the affinity for the polymer surface of the diffusing species. One approach to modelling such alterations in permeability with pressure is the Dual Mobility Model [50,61] which is described by

(11) $$K=SD_{D}+\frac{D_{H}C_{H}^{'}b}{1+bp}$$

where *S* is Henry’s constant of solubility, *D_D_* is the diffusion coefficient for species dissolved in the free volume of the polymer according to Henry’s law, *D_H_* is the diffusion coefficient for species adsorbed on the polymer molecules as described by a Langmuir isotherm, *C’_H_* is the saturation concentration of the adsorbed species and *b* (barg^−1^) is the hole affinity constant for the Langmuir adsorption sites [50]. The dual mode sorption has been described in detail by Tsujita [74].

### 1.3. Electrolyte Transport through Polymers

No published studies could be located on the transport of salts through moulded PEEK and PPS membranes. Studies on thin clay films suggest that the hydrated salt ions will permeate through the film in three steps, namely, adsorption on the interface, diffusion through the solid and desorption at the interface back into solution [75]. Similar processes will occur when salt is transported through a polymer membrane. In order to fully describe these processes, a longer experimental study with more data sets is required to validate a model. However, it is possible to make some data comparisons based on a description of some basic transport processes.

The flux (*J_i_*) across a membrane is related to the diffusion coefficient and the concentration gradient across the membrane by [68],

(12) $$J_{i}=-D_{i}\frac{\partial \;C_{i}}{\partial \;X}$$

It is important to note the meaning of the concentration gradient used in Equation (12). For a polymer film of thickness *l* separating two electrolyte solutions of concentration *C_a_* and *C_b_ (C_b_ > C_a_),* the flux of salt *J_s_* across the membrane is given by [48]

(13) $$J_{s}=\frac{D_{s}K_{s}\left(C_{b}-C_{a}\right)}{l}$$

where 𝒦_s_ is the solute partition coefficient, which is defined as the ratio of the equilibrium concentrations of salt inside and outside of the membrane (1 ≥ 𝒦_s_ ≥ 0). The product D_s_𝒦_s_ is the permeability coefficient K_s_ (Equation (2)) of the salt in the polymer membrane and if 𝒦_s_ is known for a particular polymer–electrolyte system, then the measurement of *K_s_* can be used to determine D_s_. The time dependence of the concentration difference (*C_b_ − C_a_*) is given by

(14) $$\frac{d\left(C_{b}-C_{a}\right)}{dt}=-\frac{K_{s}A\left(C_{b}-C_{a}\right)}{l}\left[\frac{1}{V_{a}}+\frac{1}{V_{b}}\right]$$

which integrates, after rearrangement, to give

(15) $$K_{s}=\frac{1}{\beta t}ln\left[\frac{{\left(C_{b}-C_{a}\right)}_{t=0}}{{\left(C_{b}-C_{a}\right)}_{t=t}}\right]$$

with the cell constant *β* given by

(16) $$\beta =\frac{A}{l}\left(\frac{1}{V_{b}}+\frac{1}{V_{a}}\right)$$

In the particular case of *V_a_ = V_b_ = V* and the initial concentration of electrolyte in one of the compartments being zero (i.e., *C_a_ = 0* at *t = 0*), the condition of negligible volume change in the two compartments during the transport of electrolyte in the polymer film gives the condition

(17) $$C_{b\left(t\right)}=C_{b\left(t=0\right)}-C_{a\left(t\right)}$$

and therefore

(18) $$K_{s}=-\frac{Vl}{2At}\ln\left[1-\frac{{2C}_{a(t)}}{C_{b(t=0)}}\right]$$

Equation (18) has a rigorous derivation for a purely diffusive process with no contributions from hydrostatic or osmotic pressure differences [76] and no significant changes in the volumes of the solutions on either side of the polymer membrane [77,78]. Yasuda et al. [35] and Geise et al. [79,80] used an identical equation to determine the permeability coefficient *K_s_* of the electrolyte in polymer membranes with no comment on its origin. Pusch [38] derived Equation (18) for the salt permeability coefficient *K_s_* in the case of a polymer membrane and dilute electrolyte solutions and noted *K_s_* and *D_s_* were related by *K_s_ =* *𝒦_s_ D_s_*. In the case when *𝒦_s_ = 1*, there is no salt rejection by the polymer membrane and the condition *K_s_ = D_s_* is obtained. 

## 2. Materials and Methods 

PPS films (Rayotec S60) were provided by Amcor, (Hawthorn Australia) as an amorphous films with an average thickness of 60 µm. Semicrystalline PPS (Fortron® 02 series, Celanese, Amsterdam, Holland) sheets with a nominal thickness of 2.3 mm were acquired from Celanese. These materials were cut into discs with a diameter of 40 mm for permeation studies. PEEK samples were supplied by Griff Paper and Film, Fallsington, USA, as semicrystalline films with an average thickness of 25 µm. Analar grade sodium chloride and potassium chloride were used. In all cases deionised water was collected in borosilicate glassware from an Elga Option S15 water purifier system. CO_2_ gas with a purity of 99.995% and a gas mixture of CO_2_ and 1.48% H_2_S were supplied in gas cylinders by CK Special Gases Ltd. 

### 2.1. Permeation Testing

Hydrogen sulfide (H_2_S) is a toxic gas and so all experiments are run in a large dedicated facility established 20 years ago at TWI headquarters near Cambridge UK. This laboratory has a central air monitoring system that alarms when low levels of toxic gases are detected. The permeation rigs are run by highly skilled and trained staff. The supply of gas mixtures containing H_2_S from gas cylinders or pumps are controlled through bespoke valves. These valves close if the central safety systems detects a H_2_S leak in the laboratory.

**Figure 1:**
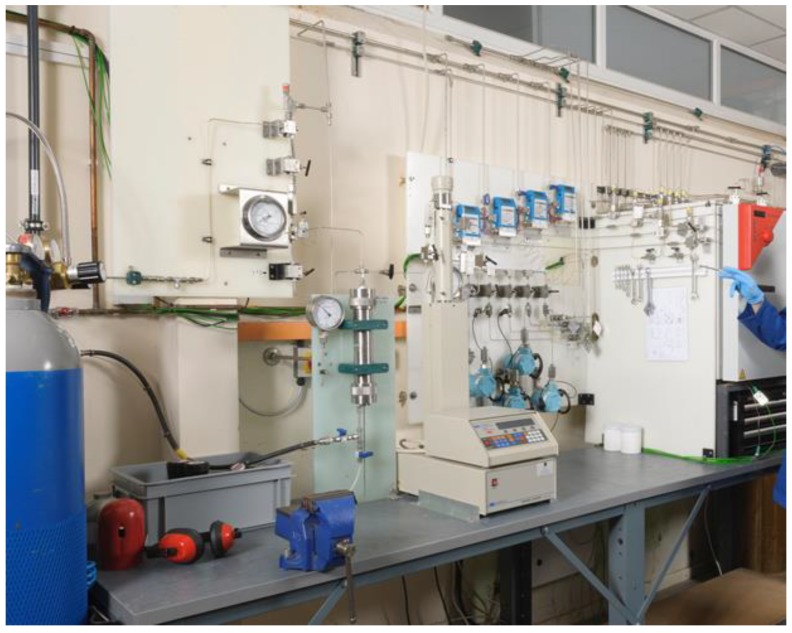
The custom-built rig used to expose polymers to supercritical CO_2_ mixtures at pressures up to 689 barg.

A photograph of the experimental rig used to pressurize gaseous CO_2_ mixtures during the preparation of supercritical CO_2_ is provided in Figure 1. 

In this circuit, a Teledyne syringe pump was used to pressurize gaseous mixtures to a maximum of 689 barg (9990 psi) as measured on the integrated pressure transducer. The system was not placed under vacuum, thus pressures are reported as bar gauge (barg). This fluid was supplied to four bespoke permeation cells housed in an oven from Binder GmBH operating in the temperature range of −10 to 300 °C. Each permeation cell contained a polymer film supported by a stainless steel sinter treated with the coating SilcoNert provided by Silco Tek. In all cases, the stainless steel sinter was continuously purged by nitrogen gas at a known flow rate of 10 mL min^−1^. The permeating gases were swept into the nitrogen-gas flow and analysed by gas chromatography. The gas chromatographs were received from PerkinElmer and modification was carried out for analysis. This permeation method is commonly known as the continuous flow permeation measurement method [81].

A disc of semicrystalline PPS was sealed onto a bespoke holder and supported by the stainless steel sinter. After purging the permeation cell with 99.995% purity nitrogen, the temperature was increased to 100 °C. Once equilibrated, one of two types of experiments was carried out. In the first variation, the experimental details are as follows:

1. CO_2_ gas containing 1.5% H_2_S was pressurised to 150 barg on the top surface of the PPS disc and the concentration of permeated CO_2_ and H_2_S measured by gas chromatography. The temperature was then reduced to 80 °C and the concentration of CO_2_ arriving at the GC was monitored.
2. The PPS disc was held at a temperature of 100 °C in the absence of test gas. Once the temperature was reduced to 80 °C, the supercritical CO_2_ containing 1.5% H_2_S was pressurised to 150 barg and exposed to the top surface of the PPS disc.
3. In another variation, CO_2_ gas was pressurized successively to 10, 50, 100, 150, 200 and 400 barg with an equilibration time allowed at each pressure step. The volume flow rate of CO_2_ permeating through the polymer film was allowed to reach a constant value before the pressure was increased to the next value. The pressure increases were achieved without the need to depressurise the cell or disturb the polymer disc.

### 2.2. Salt Transport Experiments

**Figure 2:**
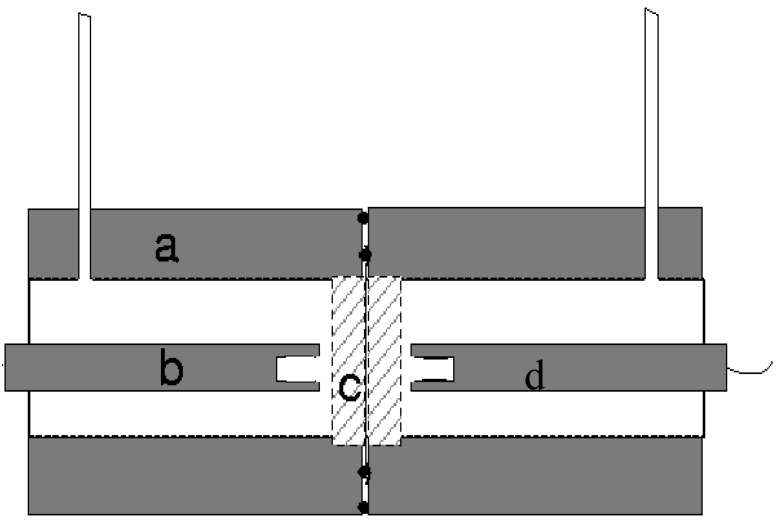
Cross-section of the osmotic cell. The cell parts highlighted are labelled (**a**) representing the cell body; (**b**) a Perspex insert; (**c**) the porous disc and (**d**) a conductivity probe. The polymer film is depicted as a fine line housed between the two discs.

**Figure 3:**
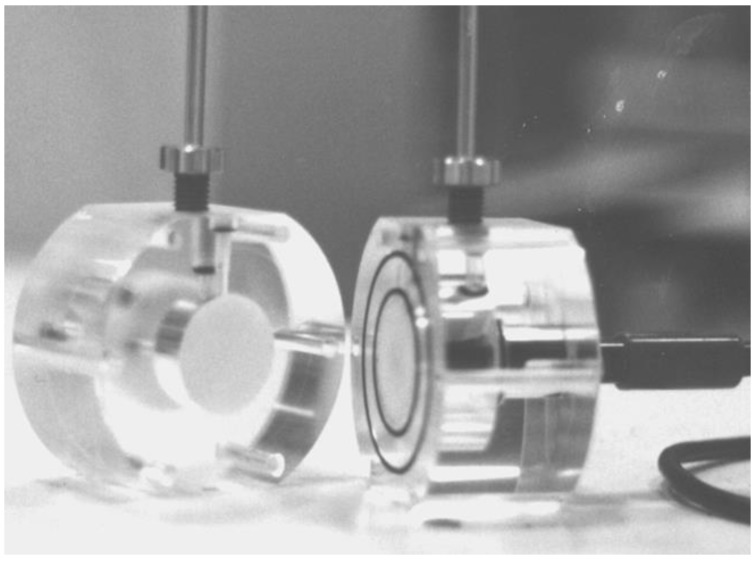
A photograph of the osmotic cell opened to reveal the porous sinters.

The transport of electrolyte and water through a thin film can be measured using a liquid diffusion cell [47]. A schematic of this cell has been provided in Figure 2 and a photograph is shown in Figure 3. The cell was machined in two halves from Perspex and a dual O-ring sealing arrangement placed in the end faces.

Porous glass filters with a diameter of 31 mm were obtained from Robu^®^ Glasfilter-Geraete and slotted into the groove in each face. The polymer film was cut to size and placed on one of the glass filters. The two cell halves were then clamped together using six threaded screws placed through the outside walls. One reservoir was filled with 0.6 M sodium chloride solution and the other with air-saturated deionised water. A glass capillary tube was connected to an inlet on either side of the polymer film. The height of the liquid in the tubes was measured allowing the pressure differential ΔP to be calculated. A conductivity probe was used to measure the electrical conductivity of the solution in the downstream chamber (low salinity chamber) in order to determine salt concentration. The conductivity probe (cell constant 0.94 cm^−1^) was monitored by a component analyser manufactured by Wayne Kerr and the salt concentration was determined using a conductivity calibration curve of the form

(19) $$\kappa =K_{1}+K_{2}C_{e}$$

where, *κ* is the electrical conductivity, *C_e_* is the concentration of salt and *K*_1_ and *K_2_* are constants. The form of Equation (19) is accurate for very dilute electrolyte solutions (0.0001 to 0.0015 molar) and therefore allows the concentration of permeating salt to be calculated as a function of time.

## 3. Results

### 3.1. Transport of CO_2_ and H_2_S through Semicrystalline PPS at Various Temperatures

**Figure 4:**
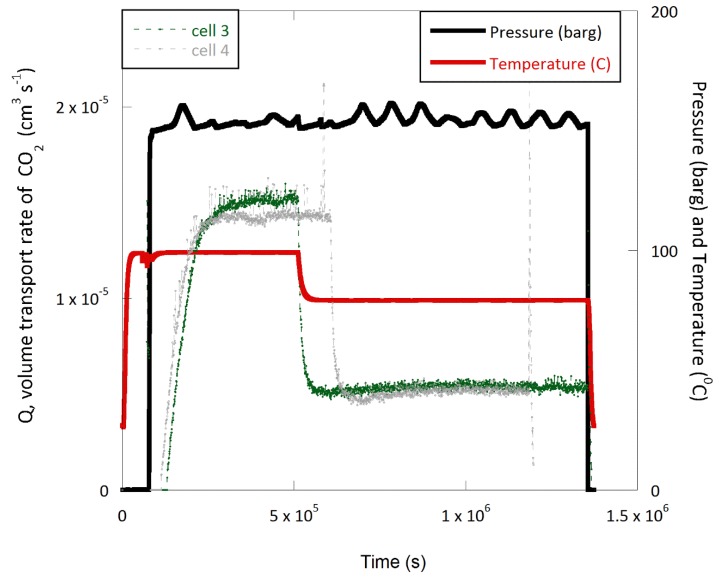
Conditioning of polyphenylene sulfide (PPS) at 100 °C followed by 80 °C; supercritical CO_2_ with 1.5% H_2_S supplied throughout in two separate experiments.

**Figure 5:**
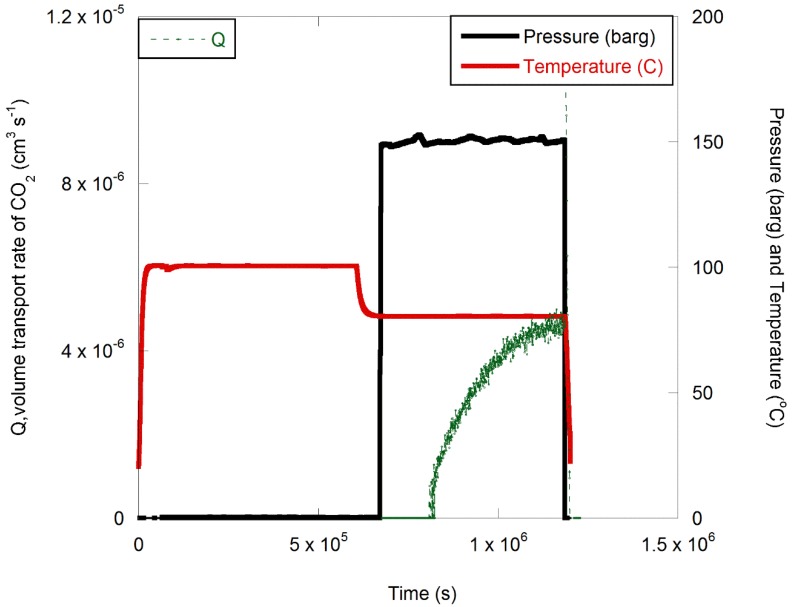
Conditioning of PPS at 100 °C followed by 80 °C; supercritical CO_2_ with 1.5% H_2_S supplied only when the temperature was reduced to 80 °C.

The profiles for the temperature, pressure and permeate composition for two types of experiments run on the experimental rig at the same time interval are presented in Figure 4 and Figure 5.

In both experiments the thermal history was the same, but in one case, the test fluid of CO_2_ with 1.5% H_2_S was supplied for the duration of the experiment both at 100 and 80 °C. In the second experiment, the test fluid was only supplied when the temperature was reduced to 80 °C, so that the effects of exposure to temperature and CO_2_ could be decoupled. The transport coefficients calculated from the permeation profiles for CO_2_ (Figure 4 and Figure 5) using Equations (1) and (5) are provided in Table 1 and Table 2. 

**Figure 6:**
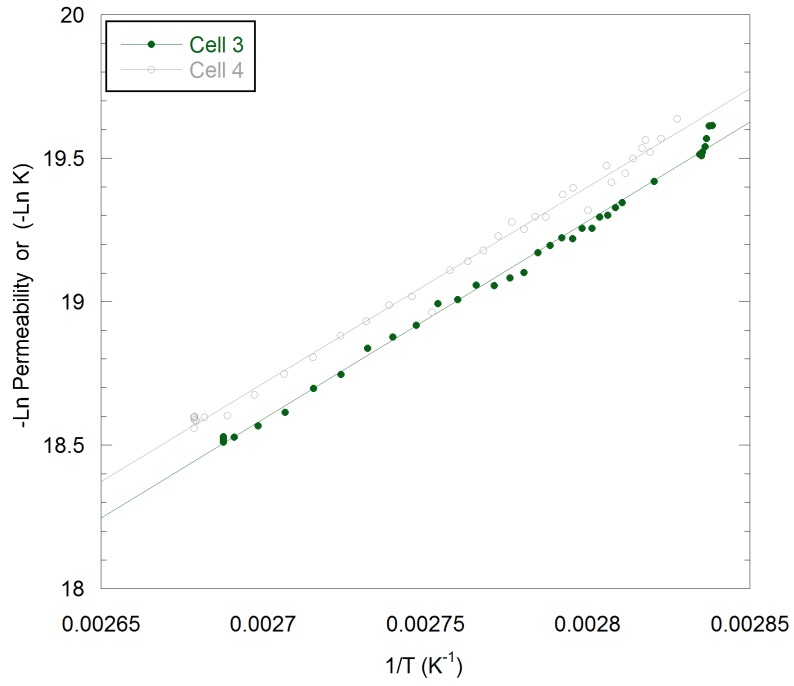
Variation in permeated levels of CO_2_ through PPS with inverse of temperature for two independent runs. Data taken from Figure 4.

Figure 6 plots the relationship between the permeability of PPS to CO_2_ and temperature. 

No H_2_S was detected in the permeate gas using gas chromatography with a limit of detection of 2 ppm, which equates to a threshold permeability of 3 × 10^−8^ (cm^2^ s^−1^ bar^−1^) at the partial pressure of H_2_S of 2.25 barg.

### 3.2. Transport of CO_2_ through Semicrystalline PPS at Various Pressures

**Figure 7:**
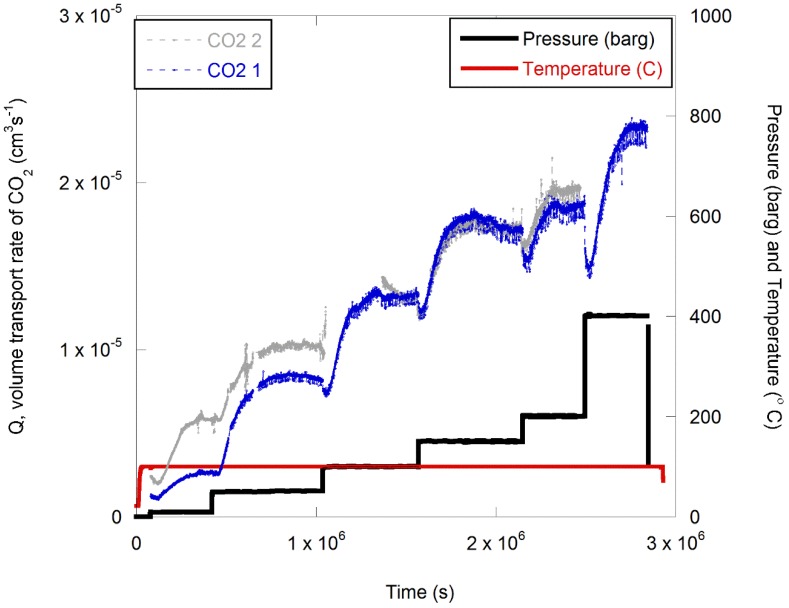
Volume transport rate of CO_2_ through PPS at 100 °C. The feed pressure was varied from 10 to 400 barg in two experimental runs.

Two experiments were run, during which pure CO_2_ was presented to the surface of a polymer film. The concentration of permeated CO_2_ as a function of the pressure is presented in Figure 7 for two separate runs. 

**Figure 8:**
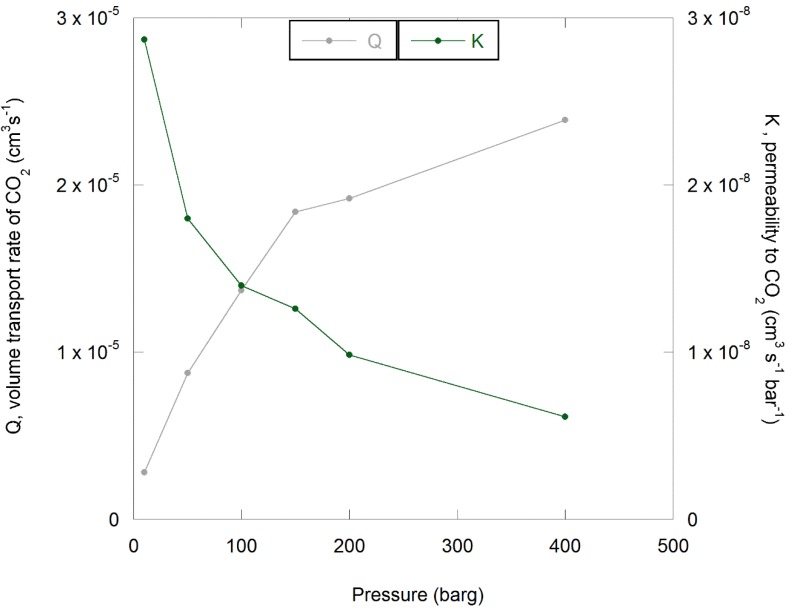
Volume flow rate (*Q*) and permeability (*K*) as a function of applied pressure for gaseous and supercritical CO_2_ conditioned at 100 °C. Data from results presented in Figure 7.

The corresponding volume flow rate (*Q*) and permeability (*K*) are plotted as a function of pressure in Figure 8. Note that failure of a seal caused spurious data at a pressure of 150 barg in one of the experiments, so data from this second run were not plotted in Figure 8. 

### 3.3. Transport of Sodium and Potassium Chloride through PPS and PEEK Films

**Figure 9:**
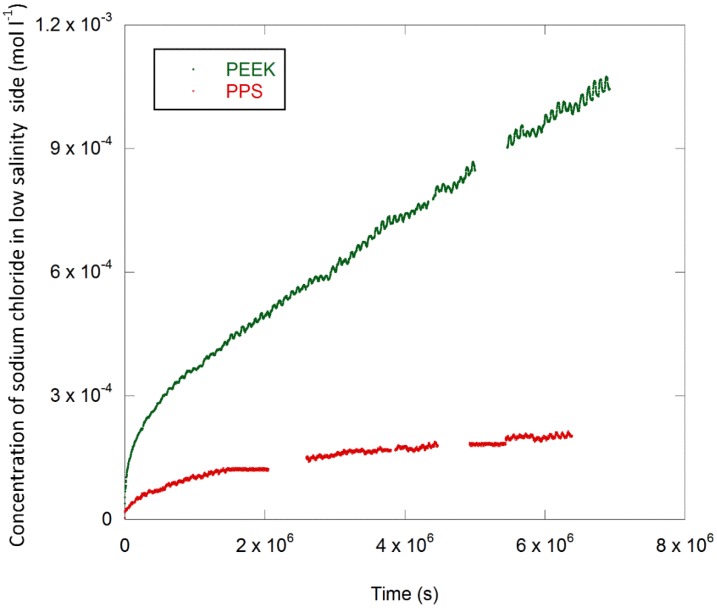
The transport of sodium chloride through PPS and polyether ether ketone (PEEK) films.

Sodium chloride concentration profiles are plotted in Figure 9. The diffusion coefficient *D_s_* of the salts was calculated using Equation (18) and the results are provided in Table 3 for both sodium chloride and potassium chloride. 

## 4. Discussion

### 4.1. Transport of CO_2_ through Semicrystalline PPS at Varying Temperature and Pressure 

The permeation experiments for the feed mixture containing supercritical CO_2_ and 1.5% H_2_S gave classical shape permeation traces for CO_2_. The partial pressure of H_2_S in the mixture was low at 2.25 barg and this alone may explain the absence of detection at the GC, as some permeation is anticipated. The absence of H_2_S could be due to a very low permeability in the polymer, which is due to either a very low diffusion coefficient or a very low solubility. The transport of CO_2_ will be discussed in the remainder of this section.

As expected from Equation (6), the permeability of CO_2_ at 100 °C was higher than when the temperature was reduced to 80 °C. The permeation level at 80 °C, as shown in Figure 4 and Figure 5, was independent of the experimental time spent exposed to CO_2_ at 100 °C. In other words, the specimens that underwent thermal ageing in the absence of CO_2_ resulted in similar permeation levels. Data listed in Table 2 and Table 3 for the permeability and diffusion coefficients indicate similar values. 

In Figure 6, it can be seen that the permeability of PPS to CO_2_ varied exponentially with T^−1^ and no discontinuity could be detected at a nominal *T*_g_ of 91 °C. Either the *T*_g_ had been reduced below 80 °C by exposure to supercritical CO_2_ or the technique was not sufficiently sensitive to changes in the glass transition temperature. Using Equation (6), the activation energy for permeation, E_a_, was determined to be 56.9 and 57.4 kJ mol^−1^ from the two data sets, which are approximately twice the value observed for PPS at 5 barg CO_2_ in the glassy state [50].

**Figure 10:**
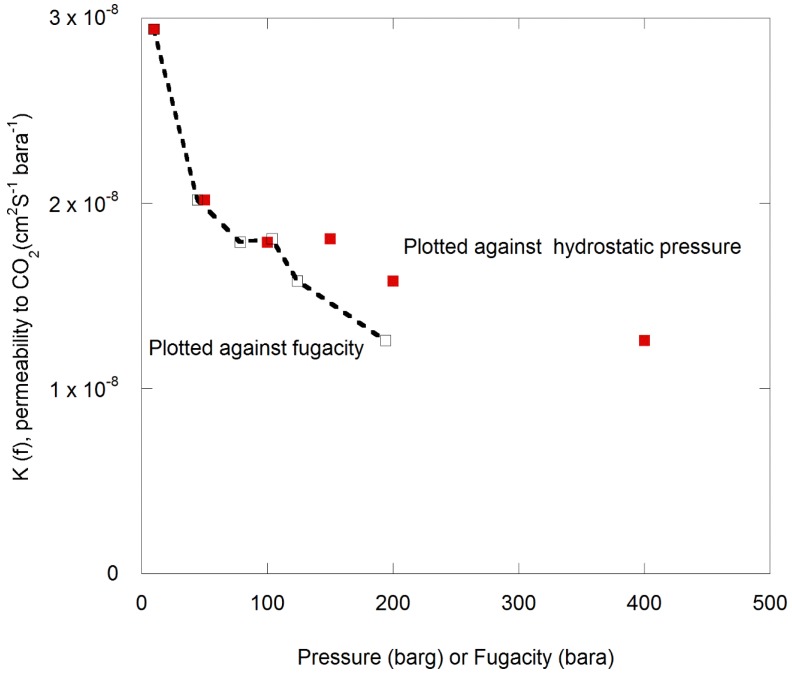
Permeability of CO_2_ through PPS calculated using fugacity plotted against hydrostatic pressure and fugacity.

The choice of transport model used to predict the barrier performance of the moulded layer depends on the response to pressure variations. In Figure 8, it can be seen that the volume flow increases linearly from the gaseous to the supercritical phase with applied pressure up to 150 barg and then the rate of increase is reduced. In this instance, the permeability was calculated using Equation (1) and was observed to decrease as the test pressure increased. Note that the permeability calculated using test fluid pressure or fugacity (deviation from ideality) also decreased with increasing pressure, as can be seen from the plot in Figure 10 [66,69]. 

**Figure 11:**
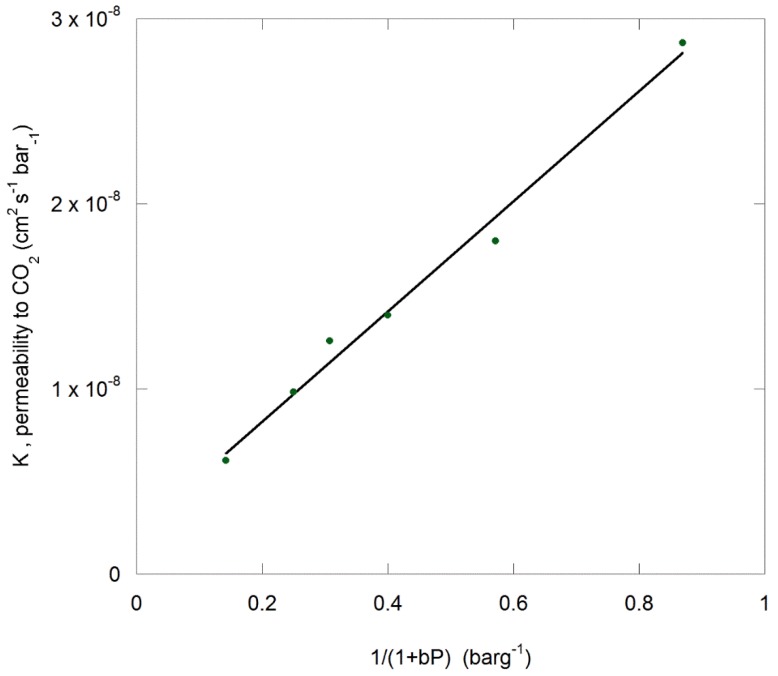
Permeability data supplied in Figure 8 for CO_2_ permeating through PPS plotted as a function of inverse pressure with a *b* value of 0.015 bar^−1^.

This deviation from linearity indicates a Langmuir-type adsorption component in addition to the Henry’s law solubility term [50,69]. In response, Equation (11) was applied to the data and a plot of K versus 1/(1+bp) is shown in Figure 11. 

The squared linear regression coefficient is 0.99 when a *b* value of 0.015 bar^−1^ is used. Independent verification of this value would require adsorption measurements to be carried out at 100 °C and pressures of 400 barg. A value for b of approximately 0.4 bar^−1^ was reported for gaseous CO_2_ adsorption on PPS in the pressure range of 40 barg at 25 °C [50]. The b value obtained for nitrogen in PPS in the pressure range of 40 barg at 25 °C by Schultze et al. [50] was in the range of 0.03 to 0.06 bar^−1^. For comparison, values of b for the permeation of nitrogen and oxygen through copoly(vinylidene cyanide-alt-vinylacetate) at 25 °C and an inlet pressure in the range 0–13.3 bar were found to vary between 0.06–0.08 bar^−1^ [74]. Further, Koros and Paul [82], determined a b value of 0.167 bar^−1^ for gaseous CO_2_ through PET at 85 °C.

The permeation profiles at varying temperature and pressures show that the plateau remains and its presence gives an indication that the PPS has not been substantially aged. 

### 4.2. Transport of Sodium Chloride and Potassium Chloride through PPS and PEEK Films

The separation of the salt solution and deionised water results in an osmotic pressure differential due to differences in the activities of the solutions on either side of the membrane. The activity coefficient of deionised water is one and that for 0.6 M NaCl solution is 0.980, [83], whereupon the value of the osmotic pressure differential ∆π is expected to be 27.8 bar at 25 °C. 

Essentially, the salt diffuses from high to low salinity and brings water with it in the solvation shell, while the free water diffuses from the low salinity across the polymer film to the high salinity side. All species, where possible, will move until their chemical potentials on either side of the polymer film are equal. Water will continue to move into the high salinity side of the reservoir, as this is the area with low water concentration (low water activity). The diffusion coefficient for sodium chloride NaCl and potassium chloride KCl at infinite dilution and 25 °C are 1.6 and 1.9 × 10^−5^ cm^2^ s^−1^, respectively [84]. This difference is due in part to the relative size of the hydrated cation radius, namely 0.66nm for K^+^ and 0.72 nm for Na^+^ [85]. The diffusion coefficients of sodium and potassium chloride calculated from these permeation studies are in the range of 0.9 to 9.7 × 10^−12^ cm^2^ s^−1^.

For comparison, the diffusion coefficient of sodium chloride was determined to be 4 × 10^−11^ cm^2^ s^−1^ for HDPE at 23 °C [33], while a value of 6–8 × 10^−11^ cm^2^ s^−1^ was determined for sodium chloride at 20 °C [86]. The diffusion coefficient for sodium chloride through Teflon was determined as being between 2 and 3 × 10^−11^ cm^2^ s^−1^ [79]. In addition, Aithal et al. [45] determined a diffusion coefficient of sodium chloride in NBR and EPDM at 23 °C of 4.8 × 10^−14^ cm^2^ s^−1^ and 4.1 × 10^−14^ cm^2^ s^−1^, respectively. 

The water was detected to have moved across the PPS and PEEK films into the high salinity side. The hydrostatic pressure differentials developed were on the order of kPa, which is indicative of coupled osmotic flow [47].

## 5. Conclusions

Novel experimental apparatus and methodologies have been developed to quantify the transport of CO_2_ and chloride ions through polymer membranes under varying conditions of temperature (up to 100 °C) and pressure (up to 690 bar) and for prolonged periods of time. The permeated gas mixture was analysed by gas chromatography. 

Typical measurement procedures were demonstrated with PPS with a feed gas of supercritical CO_2_ and 1.5% H_2_S. The polyphenylene sulfide (PPS) membrane used was stable upon exposure to supercritical CO_2_. No evidence of the glass transition temperature was found during the temperature sweep from 100 to 80 °C. The activation energy for permeation was calculated as 54 kJ mol^−1^, which is approximately twice the published value observed for PPS at 5 barg CO_2_ in the glassy state. An increase in the feed pressure of pure CO_2_ resulted in a decrease in the permeability of the PPS samples, indicating the presence of a Langmuir-type adsorption process. In future research, this Langmuir fit can be validated further by the creation of adsorption isotherms using a recently acquired High Pressure Thermogravimetric analyser from Rubotherm. The permeation of H_2_S through PPS was not detected. 

The diffusivities of sodium chloride and potassium chloride in PEEK are higher than the corresponding values in PPS. The diffusion coefficient of potassium chloride is higher than sodium chloride in both PPS and PEEK.

## Figures and Tables

**Table 1:** Transport coefficients of CO_2_ at 150 barg through PPS.

Temperature (°C)	100	80
Permeability (K) 10^−9^ (cm^3^ (STP) cm^−1^ s^−1^ bar^−1^)	8.6	3.0
Diffusion coefficient (D) 10^−8^ (cm^2^ s ^−1^)	7.3	^1^

^1^ cool down eliminates initial flux rise and prevents calculation of D.

**Table 2:** Transport coefficients of CO_2_ at 150 barg through PPS.

Temperature (°C)	100	80
Permeability (K) 10^−9^ (cm^3^ (STP)cm^−1^ s^−1^ bar^−1^)	No fluid supplied	2.8
Diffusion coefficient (D) 10^−8^ (cm^2^ s ^−1^)	No fluid supplied	2.8

**Table 3:** Diffusion coefficients for sodium chloride and potassium chloride in PEEK and PPS under ambient conditions.

Polymer	Salt	Thickness (µm)	Diffusion Constant 10^−12^ (cm^2^ s^−1^)
PEEK	NaCl	25	0.9
PPS	NaCl	60	0.4
PEEK	KCl	25	7.9
PPS	KCl	60	1.2
